# Overexpression of the Vitronectin V10 Subunit in Patients with Nonalcoholic Steatohepatitis: Implications for Noninvasive Diagnosis of NASH

**DOI:** 10.3390/ijms19020603

**Published:** 2018-02-18

**Authors:** Maria Del Ben, Diletta Overi, Licia Polimeni, Guido Carpino, Giancarlo Labbadia, Francesco Baratta, Daniele Pastori, Valeria Noce, Eugenio Gaudio, Francesco Angelico, Carmine Mancone

**Affiliations:** 1 Department of Internal Medicine and Medical Specialties, Sapienza University of Rome, Viale del Policlinico 155, 00161 Rome, Italy; maria.delben@uniroma1.it (M.D.B.); licia.polimeni@uniroma1.it (L.P.); giancarlo.labbadia@uniroma1.it (G.L.); francesco.baratta@uniroma1.it (F.B.); daniele.pastori@uniroma1.it (D.P.); 2 Department of Anatomical, Histological, Forensic Medicine and Orthopedics Sciences, Sapienza University of Rome, Via Borelli 50, 00161 Rome, Italy; diletta.overi@uniroma1.it (D.O.); eugenio.gaudio@uniroma1.it (E.G.); 3 Department of Movement, Human and Health Sciences, Division of Health Sciences, University of Rome “Foro Italico”, Piazza Lauro De Bosis 6, 00135 Rome, Italy; guido.carpino@uniroma1.it; 4 Department of Cellular Biotechnologies and Haematology, Sapienza University of Rome, Viale del Policlinico 155, 00161 Rome, Italy; noce@bce.uniroma1.it; 5 Department of Public Health and Infectious Diseases, Sapienza University of Rome, Viale del Policlinico 155, 00161 Rome, Italy; francesco.angelico@uniroma1.it

**Keywords:** nonalcoholic fatty liver disease, nonalcoholic steatohepatitis, liver fibrosis, secretome

## Abstract

Nonalcoholic steatohepatitis (NASH) is the critical stage of nonalcoholic fatty liver disease (NAFLD). The persistence of necroinflammatory lesions and fibrogenesis in NASH is the leading cause of liver cirrhosis and, ultimately, hepatocellular carcinoma. To date, the histological examination of liver biopsies, albeit invasive, remains the means to distinguish NASH from simple steatosis (NAFL). Therefore, a noninvasive diagnosis by serum biomarkers is eagerly needed. Here, by a proteomic approach, we analysed the soluble low-molecular-weight protein fragments flushed out from the liver tissue of NAFL and NASH patients. On the basis of the assumption that steatohepatitis leads to the remodelling of the liver extracellular matrix (ECM), NASH-specific fragments were in silico analysed for their involvement in the ECM molecular composition. The 10 kDa C-terminal fragment of the ECM protein vitronectin (VTN) was then selected as a promising circulating biomarker in discriminating NASH. The analysis of sera of patients provided these major findings: the circulating VTN fragment (i) is overexpressed in NASH patients and positively correlates with the NASH activity score (NAS); (ii) originates from the disulfide bond reduction between the V10 and the V65 subunits. In conclusion, V10 determination in the serum could represent a reliable tool for the noninvasive discrimination of NASH from simple steatosis.

## 1. Introduction

Nonalcoholic fatty liver disease (NAFLD) is by now the most common liver disease in the developed and developing countries with a global estimated median prevalence of 20% [1]. Most NAFLD patients have simple steatosis or nonalcoholic fatty liver (NAFL) with low inflammation, tissue damage, or liver fibrosis. However, 13% to 31% of the cases develop definite nonalcoholic steatohepatitis (NASH), characterized by hepatic steatosis and inflammation with ballooning, with or without fibrosis [2]. The prognosis is poorer in patients with NASH, and NASH patients can progress to liver cirrhosis and ultimately hepatocellular carcinoma (HCC) [3]. Therefore, it is crucial to obtain a prompt diagnosis of NAFLD patients with NASH for an effective clinical management.

Currently, the differentiation of NAFL from definite steatohepatitis and liver fibrosis in the whole spectrum of NAFLD is still based on the histological examination of liver biopsies to assign a grade and stage through scoring systems [4]. The NAFLD Activity Score (NAS) is widely accepted and used in clinical practice [5]. To stage fibrosis, the NASH Clinical Research Network system is one of the most validated systems currently available and defines four fibrosis stages [5,6]. Nevertheless, although the histological evaluation of liver biopsies addresses the full spectrum of lesions of NAFLD, this procedure remains invasive and limited by sampling error, diagnostic accuracy, and hazard to the patients [7].

Serum biomarkers offer a noninvasive and cost-effective alternative to liver biopsy both for patients and clinicians. Previous studies on NAFLD subjects described some circulating proteins and metabolites with a potential for discriminating between NASH and NAFL [8,9,10,11]. Nevertheless, the feasibility, specificity, and sensitivity of these candidate biomarkers are relatively low or, in some cases, only reflect one aspect of NAFLD progression instead of the overall condition. There is, therefore, an increasingly pressing need to identify NAS-related serum biomarkers that can reflect the grade of the disease. In this regard, low-abundance peptides in the serum may represent a reservoir of NASH-specific products originating from leakage as a result of cell death, extracellular matrix remodelling, or damages [12]. Unfortunately, one of the challenges in proteomic-based serum marker discovery is that serum samples are dominated by high-abundance proteins whose presence obscures less abundant products. In particular, the high concentrations of albumin and immunoglobulins (Igs) in serum samples prevent the successful identification of low-abundance biomarkers in studies based on top-down peptidomic approaches. Moreover, methods developed for depleting the high-abundance proteins, while eliminating the most of albumin and Igs, lead to the impoverishment of minor products. Therefore, identifying potential circulating markers of NASH straight from serum samples still remains an ambitious challenge.

In this study, to overcome this issue, we firstly performed a proteomic analysis of soluble low-molecular-weight (LMW) polypeptides flushed out from liver biopsies of NAFLD patients. The 10 kDa subunit (V10) of vitronectin was identified as upregulated in the flush-out samples of the NASH liver sample group. Hence, our aim has been to provide a proof of concept regarding the possible correlation of serum vitronectin fragments with histopathology in patients affected by NAFLD and its putative role in the assessment of disease severity.

## 2. Results

### 2.1. Characteristics of the Patients

The group of NAFLD patients included 50 subjects (24 males and 26 females). Among them, 27 subjects did not present definite steatohepatitis (NAFL group) while 23 subjects displayed definite steatohepatitis (NASH group). The fibrosis stage in NASH patients was more advanced than in those affected by NAFL. The patient’s age did not statistically differ between NAFL and NASH groups. The body mass index (BMI), aspartate aminotransferase (AST), and alanine aminotransferase (ALT) levels were significantly higher in subjects with NASH than in those with NAFL. Gamma-glutamyltransferase (GGT) levels and AST/ALT ratio were not significantly different between the groups. The clinical and serological characteristics of the patients are summarized in Table 1.

### 2.2. Identification of LMW Polypeptides in NAFL and NASH Samples

The LMW polypeptides released from the liver tissue are an essential part of the NASH microenvironment and represent a reservoir of early promising and specific biomarkers firstly detectable in the NASH-specific secretome and, subsequently, circulating in the blood as low-abundance products. Therefore, a comparative proteomic analysis of enriched LMW polypeptides flushed out from the liver biopsies of NAFLD patients could represent a powerful alternative strategy for the discovery of circulating subnanomolar biomarkers.

**Figure 1:**
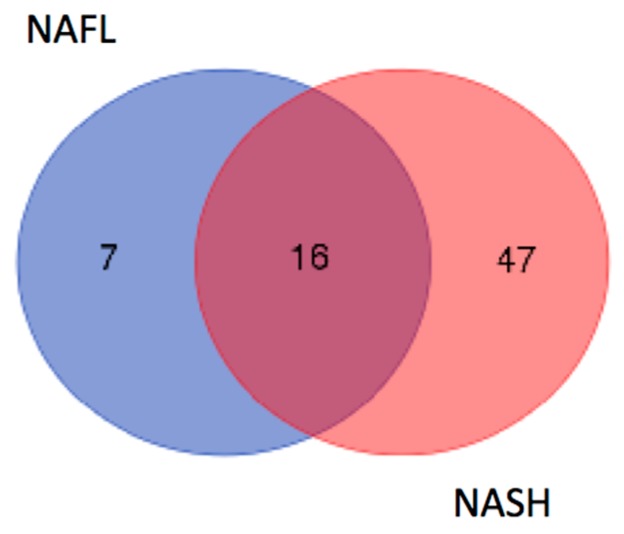
Venn diagram of the overlap of identified protein fragments in NASH and NAFL groups.

Secretomes from the liver biopsies were individually collected, and equal amounts of proteins from patients having a diagnosis of simple steatosis (NAFL group) and definite steatohepatitis (NASH group) were pooled. One hundred micrograms of proteins from each pool was separated by non-reducing SDS-PAGE with a buffer system specific for the resolution of low-molecular-weight polypeptides. Each gel lane was cut into seven sections ranging from 3 to 20 kDa, and the polypeptides in each gel section were digested and submitted to nLC–MS-based proteomic analysis. A total of 235 and 119 proteins in NASH and NAFL groups, respectively, were identified (Table S1). Aiming at selecting peptides originating from the fragmentation of whole proteins, we used the electrophoretically derived molecular weight (MWexp) of the protein as the identification constraint. On the basis of the assumption that the MWexp of a protein should correlate with its theoretical molecular weight (MWcal) obtained from the database search, 63 and 23 identifications resulted as protein fragments in NASH and NASL groups, respectively (Table S1). Interestingly, by comparing these two datasets, 7 protein fragments were identified in the NAFL group, 16 were commonly identified, and 47 were found exclusively in the NASH group, thus confirming that the disease progression leads to increased protein fragmentation (Figure 1).

### 2.3. In Silico Analysis of NASH-Specific Fragments

In response to inflammatory stimuli, NASH leads to changes in the composition of the liver extracellular matrix (ECM), where high levels of ECM protein fragments are generated and released into the circulation at low concentrations [13]. Therefore, it is conceivable that liver-specific ECM circulating peptides may be the one of the most suitable candidates as circulating NAS-related biomarkers for assessing grade and stage in NASH patients.

The 47 proteins, whose fragments were found exclusively in the NASH group, were then analysed for their involvement in the ECM molecular composition by interrogating the MatrisomeDB 2.0 (http://matrisomeproject.mit.edu/proteins/), a searchable database that integrates experimental proteomic data on ECM and ECM-associated proteins from the ECM Atlas [14]. As shown in Table 2, seven proteins were found to be associated with liver ECM, five of which as minor components or regulators (i.e.: LGALS3, LGALS4, CTSB, SERPINB1, SERPINC1), and the vitronectin (VTN) and fibrinogen alpha chain (FGA) as main structural components. Since several reports demonstrated that the VTN is upregulated in the ECM of fibrotic liver [15,16,17,18], it is reasonable to hypothesize that this protein may be subjected to turnover and degradation. Accordingly, we decided to challenge the detection of the VTN fragment directly in the sera of patients.

### 2.4. Correlation between Circulating VTN Fragments and Liver HistoMorphology

**Figure 2:**
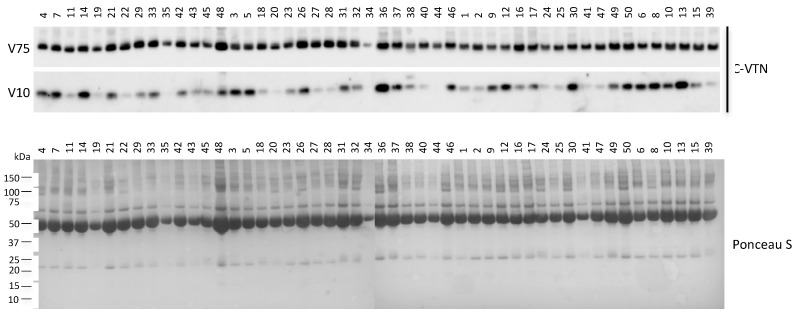
Western blotting analysis of 10 kDa VTN fragment levels in NAFLD patients. Dilutions (1:10) of the serum samples were separated on a non-reducing SDS polyacrylamide gel and probed with an antibody specific for the vitronectin C-terminal end (C-VTN). Total protein staining by Ponceau S on the nitrocellulose membrane is shown. Patient IDs are indicated. These images are representative of experiments carried out in triplicate

**Figure 3:**
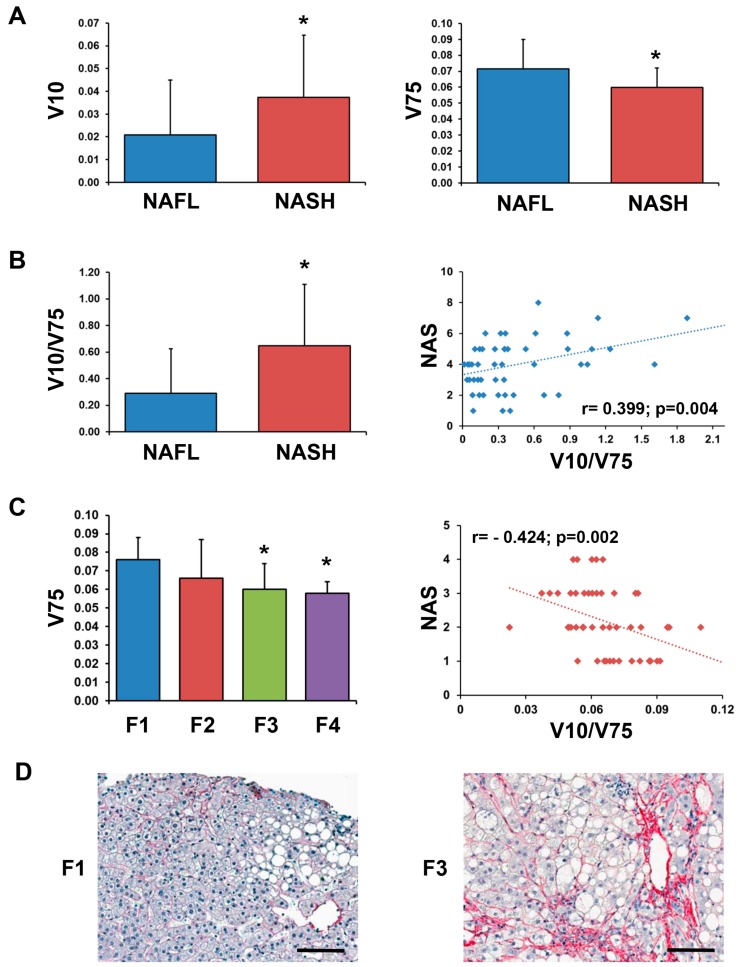
V10 and V75 levels are increased in NASH patients’ serum. (**A**) Densitometry analysis of the V10 and V75 blots imaged in Figure 2. The total lane density detected from the Ponceau S staining of the transferred protein in the blots was used for normalization. Patients with definite steatohepatitis (NASH group) have higher V10 and lower V75 levels compared with patients without definite steatohepatitis (NAFL group); * = *p* < 0.05. (**B**) Histogram showing that the V10/V75 ratio was higher in NASH compared to NAFL group (left); * < 0.05. The scatter plot graph on the right shows the correlation between the V10/V75 ratio and the NAS score. (**C**) The histogram on the left shows V75 serum levels in patients divided according to the fibrosis (F) score; * < 0.01 versus F1 group. The scatter plot graph on the right shows the correlation between V75 levels and the fibrosis score. (**D**) Sirius red stains in liver biopsies are representative of F1 and F3 stages, respectively. Scale bar = 100 µm.

Mature vitronectin is a multifunctional plasma and extracellular matrix protein of 459 amino acids with a MWcal of 52 kDa; the observed MWexp of the glycosylated form is 75 kDa [19]. We identified the VTN fragment in the 12–7 kDa SDS-PAGE-displayed molecular weight range. Interestingly, by tandem mass spectrometry analysis, we found that the sequence of the tryptic peptides, originated from the digestion of the VTN fragment, matched with the C-terminal end of the protein (Table S2). To detect this fragment in the sera of our cohort of patients, a non-reducing western blotting analysis was performed by using an antibody that recognizes the vitronectin C-terminal end. As expected, the 75 kDa form of VTN (V75) was detected in the serum of NAFLD patients (Figure 2). Moreover, an additional signal of approximately 10 kDa (V10) was detected, thus confirming the existence of a circulating C-terminal fragment. Surprisingly, the V10 level of expression did not reflect the expression level of the circulating mature form. We then analysed the serum levels of V10 and V75 in the frame of NASH by densitometry. As shown in Figure 3, NASH patients displayed increased levels of V10 compared to the NAFL group (*p* = 0.027). On the contrary, lower amounts of V75 were measured in the serum of NASH patients (*p* = 0.013) and in patients with a fibrosis score >2 compared to patients with a fibrosis score =1 (*p* < 0.01). Then, we further extended the analysis by using the V10/V75 ratio (Figure 3). Interestingly, the V10/V75 ratio was significantly higher in NASH compared to NAFL patients (*p* = 0.003). By bivariate analysis (Figure 3), V10, V75, and V10/V75 ratio significantly correlated with the NAS score (respectively: *r* = 0.311, *p* = 0.028; *r* = −0.318, *p* = 0.024; *r* = 0.399, *p* = 0.004) but not with clinical–serological parameters (i.e., BMI, ALT, AST); only the V75 fragments correlated with the fibrosis score (*r* = −0.424; *p* = 0.002). To further assess whether these results could be actually due to the reported differences in BMI, ALT, and AST between the two groups (NAFL and NASH), a linear regression analysis was performed by two models using NAS or, alternatively, fibrosis as independent variables. The ratio V10/V75 resulted a predictor of NAS (beta = 0.354; *p* = 0.010) but not of fibrosis (beta= 0.166; *p* = 0.248), independently from the above-mentioned parameters (i.e., BMI, ALT, AST); moreover, the V75 fragment resulted a predictor of fibrosis (beta= −0.292; *p* = 0.034) but not of NAS (beta= −0.279; *p* = 0.051), independently from BMI, ALT, AST. Given the low beta value, we further tried to individuate a possible cut-off value for the V10/V75 ratio by analysing sera from healthy subjects (*N* = 6). The V10, V75, and V10/V75 ratio values resulted significantly higher in NAFLD patients compared to healthy subjects (*p* < 0.05). For the V10/V75 ratio, we assumed the highest value obtained in healthy subjects as a possible cut-off (0.27) to be used as a threshold for NAFLD patients. The binary logistic regression demonstrated that NASH diagnosis was associated with a V10/V75 ratio over (>0.27) the identified threshold (OR: 5.254; CI 95%: 1.142–24.163; *p* = 0.033) after adjustment for BMI, AST, ALT.

### 2.5. Circulating V10 Originates by the Reduction of VTN Clipped Form

VTN is produced and secreted by the hepatocytes in two molecular forms of 75 kDa: a single, non-cleaved chain and a furin-mediated clipped form composed of two chains of 65 and 10 kDa which are held together by a disulfide bond [19]. Consequently, we wondered whether the increased level of the C-terminal fragment of vitronectin in the sera of patients with NASH may be related to the degradation of vitronectin by proteases overproduced in NASH or to the reduction of the disulfide bond between the two vitronectin subunits.

**Figure 4:**
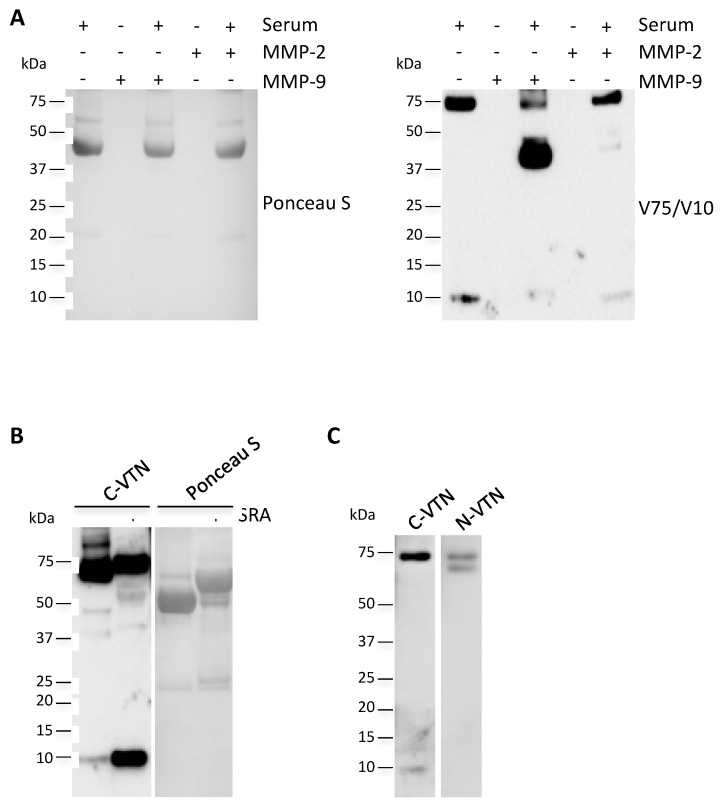
The 10 kDa VTN fragment originates from the reduction of the disulfide bond between the V65 and V10 subunits. (**A**) C-VTN non-reducing western blotting analysis of 1:10 dilutions of a NASH serum sample in the presence or absence of MMP-2 and -9. Total protein staining by Ponceau S on the nitrocellulose membrane is shown. (**B**) C-VTN western blotting analysis of 1:10 dilutions of a NASH serum sample in the presence or absence of a sample-reducing agent (SRA). Total protein staining by Ponceau S on the nitrocellulose membrane is shown. (**C**) Non-reducing western blotting analysis of 1:10 dilutions of a NASH serum sample probed with antibodies specific for the vitronectin C-terminal (C-VTN) and N-terminal (N-VTN) ends. These images are representative of experiments carried out in triplicate.

The degradation of vitronectin occurs by means of matrix metalloproteinases (MMPs)-1, -2, -3, -7, and -9 [20]. Since MMP-2 and -9 are found extensively upregulated in NASH [21], we first sought to determine whether in vitro digestion of circulating VTN by these MMPs gave rise to increased levels of V10. As indicated in Figure 4A, the proteolytic activity generated fragments ranging from 37 to 45 kDa. Nevertheless, the 10 kDa signals were found at lower levels with respect to the control, thus suggesting that the MMP-2 and -9 are not responsible for the V10 production.

Then, we verified the hypothesis that V10 originated by the releasing of the 10 kDa subunit from the vitronectin clipped form by means of disulfide bond reduction. Firstly, we proved that the addition of a reducing agent generated a signal corresponding to the same molecular weight of V10 (Figure 4B). Then, we analysed the presence of the free circulating 65 kDa subunit (V65) by using an antibody that recognizes the vitronectin N-terminal end. As reported in Figure 4C, in addition to the 75 kDa signal, the immunoblot after non-reducing electrophoresis highlights the presence of a further band corresponding to the V65 subunit, thus suggesting that the circulating 10 kDa fragment could be the result of the disulfide bond reduction.

## 3. Discussion

Our analysis on sera of NAFLD patients showed that: (i) circulating V10 is overexpressed in NASH patients compared to NAFL patients and positively correlates with NAS; (ii) circulating V75 is underexpressed in NASH patients compared to NAFL patients and negatively correlates with NAS and liver fibrosis; (iii) the V10/V75 ratio is a predictor of NAS score, independently from BMI, ALT, and AST; (iv) circulating V10 originates from the disulfide bond reduction of the V75 clipped form.

The aim of the present study was to identify circulating serum markers which could be helpful in distinguishing patients with high NAFLD activity or with advanced fibrosis. For this purpose, we focused our attention on those peptides originated by the fragmentation of mature proteins that may reflect valuable disease-related information. However, the proteomic screening for these fragments was performed on liver biopsy secretomes to overcome complexity and detection problems associated with peptide analysis of serum or plasma. Serum and plasma proteins are present at concentrations that are likely to extend over 10 orders of magnitude [22]. In addition, the 22 major proteins, including albumin and immunoglobulins, representing 99% of total serum polypeptides, are preferentially sequenced by mass spectrometry [23]. Therefore, the identification of the remaining 1%, consisting of thousands of LMW proteins and peptides, remains challenging despite the proposed strategies for peptide extraction from serum [24,25]. Here, by enriching LMW polypeptides flushed out from the liver specimens of patients, we generated NAFL- and NASH-specific data sets of LMW protein fragments whose comparison highlighted an increased tissue protein turnover during the progression of the disease from the simple steatosis to the steatohepatitis. To gain insights on the specific valence of the protein fragments found in NASH, we focused our attention on the liver ECM specific products which may reflect the pathological matrix remodelling in steatohepatitis. Five ECM-derived fragments were found, and, among these, we restricted the experimental observations to the vitronectin 10 kDa C-terminal fragment.

Vitronectin has been extensively studied in the frame of liver fibrosis in chronic liver diseases such as viral hepatitis B and C infections and HCC. Particularly, the ECM-associated vitronectin is found at low concentrations in normal liver and markedly increases in the cirrhotic liver, while the circulating 75 kDa form follows an opposite trend [15,16,17,18].

Here, for the first time, we analysed the expression of circulating VTN in the frame of NAFLD. We demonstrated that in NASH patients, VTN undergoes molecular remodelling that releases the two subunits from the clipped form. Since the small subunit V10 was found at high concentrations in NASH, while the V75 form was found to be decreased, the ratio V10/V75 might be a suitable serological indicator helpful for discriminating the presence of steatohepatitis in NAFLD patients. A 10 kDa vitronectin C-terminal fragment has been previously identified as a serum marker of HCC [26]. Nevertheless, while in HCC the V10 is produced by the catalytic activity of MMP-2 on the V75 non-cleaved form, we demonstrated that in NASH it originates from the disulfide bond reduction of the clipped form. Oxidative stress has been extensively demonstrated to be a major factor in the development of NASH [27,28,29]. Particularly, it has been shown that the production of reactive oxygen and nitrogen species in a context of liver steatosis promotes lipid peroxidation which, in turn, supports the necroinflammatory milieu and leads to the stimulation of collagen synthesis in hepatic stellate cells [30,31]. To counteract the oxidative stress, mammalian cells activate thioredoxin (Trx), which maintains a reducing environment by catalysing an electron flux from nicotinamide adenine dinucleotide phosphate to Trx through Trx reductase, which reduces its target proteins using highly conserved thiol groups [32]. Therefore, it is conceivable that the oxidative stress-induced elevated serum [33] and tissue (Table S1) Trx levels in NASH patients may be responsible for the free V10 production. Moreover, since increased hepatic vitronectin expression favors fibrogenesis by recruiting lymphocytes within the inflamed liver tissue and promoting a wound-healing response [19,34], the oxidative stress-induced reduction of the functional V75 clipped form may be the consequence of a pathophysiological response for counteracting liver fibrosis and the disease progression. Further experiments may challenge the robustness of these insights.

The validation and clinical availability of serum biomarkers of NASH are desirable to aid clinicians in the discrimination of NAFLD patients with steatohepatitis from simple steatosis and for the noninvasive monitoring of disease progression and response to therapy. To date, the most promising proposed serum biomarkers (i.e.: cytokeratin-18 M30 fragment [8], fibroblast growth factor 21 [35], interleukin 1 receptor antagonist [36], pigment epithelium-derived factor [11], osteoprotegerin [37]), showed a potential in diagnosing NAFLD and NASH. However, taken individually, they reflect only one aspect of the NAFLD pathological scenery, i.e., hepatocyte apoptosis, inflammation, fibrosis, insulin sensitivity, and steatosis. Moreover, some of them, showing low sensitivity and accuracy, need to be validated in a larger cohort [38]. However, it has been demonstrated that tests performed by combining these biomarkers improved the accuracy in the diagnosis of NASH [38]. Thus, we believe that the measurement of V10 in blood could be a further useful tool for detecting NASH by means of a panel of markers. However, the present study represents a proof of concept regarding the possibility to detect vitronectin fragments in the serum of patients affected by NAFLD and a possible correlation with histomorphology. Thus, our results should be replicated in a larger cohort of NAFLD patients to validate their eventual clinical relevance. In addition, it has, in general, several limitations: (i) a sampling bias may have originated as liver biopsy was used as gold standard for assessing the use of V10; (ii) the samples were all selected within the Italian population; (iii) the lack of validation based on a gel-free quantitative immunoassay able to discriminate the C-terminal V10 from the V75; (iv) the specificity of the V10 fragment in relation to hepatocyte injury in NASH has not been challenged in patients with other liver diseases. Moreover, since the presence of threonine rather than methionine at position 381 was proposed to be responsible for the susceptibility of VTN to cleavage at Arg^379^–Ala^380^ for subunits production [19], a genetic SNP analysis of the VTN gene may be considered for those NASH patients with low levels of circulating V10.

In conclusion, our findings suggest that the circulating VTN 10 kDa subunit may be a reliable tool in the discrimination of patients with NASH.

## 4. Materials and Methods

### 4.1. Patient Characteristics

The study was approved by the Institutional Ethic Committee of Sapienza University of Rome (prot. 873/11, Rif. 2277 approved on 13 October 2011) and conforms to the ethical guidelines of the 1975 Declaration of Helsinki. The population for the current study consisted of 50 well-characterized, biopsy-proven adult NAFLD patients. To be eligible for the study, NAFLD patients had to fulfill the following criteria: ultrasound evidence of fatty liver (defined according to Hamagouchi criteria), absence of current or past excessive alcohol drinking as defined by an average daily consumption of alcohol >20 g for women and >30 g for men; negative tests for the presence of hepatitis B surface antigen and antibody to hepatitis C virus. Percutaneous liver biopsy was performed under ultrasound guidance in fatty liver patients with clinical suspicion of NASH by their treating hepatologists. The decision to perform the biopsy was individualized and based on a persistent elevation of serum alanine aminotransferase levels (>1.5 above normal values) for more than 6 months and the presence of risk factors for NASH. A single operator performed ultrasound-guided liver biopsies. The pathologist who examined the biopsy specimens was blinded to patients’ identity or clinical information. Diagnosis of definite steatohepatitis (i.e., NASH) was defined using standard criteria [4], and NAFLD activity score (NAS) was calculated on the basis of separate scores for steatosis, hepatocellular ballooning, and inflammation [5]. Fibrosis was scored on a scale of 0–4 [5]. Blood samples from six control (healthy) subjects was analysed. Control subjects (three male and three female) with age = 49 ± 13 years (mean ± standard deviation) were included; these subjects had a BMI < 25, did not have metabolic risk factors, (e.g., diabetes, hypertension), were negative for viral hepatitis markers, had normal liver serological tests, and had no sign of liver steatosis at ultrasound. A written informed consent was obtained from all subjects.

### 4.2. Liver Secretome, Protein Digestion, and Peptide Purification

Secreted proteins from fresh liver biopsies were obtained by overnight shaking (600 RPM) in Washing Buffer (0.5 M NaCl, 10 mM Tris Base pH 7.5, 1× protease inhibitor cocktail (Sigma Aldrich, St. Louis, MO, USA)) at 37 °C. After centrifugation at 13,000 rpm for 1 min, the supernatants were collected, and protein concentrations were measured by Bradford assay. Equal amounts of samples were individually collected to generate pools of 100 μg. The samples were then separated on 12% gels (Life technologies, Thermo Fisher Scientific, Waltham, MA, USA) by SDS-PAGE with MES running buffer (Life technologies). The gels were stained by Simply Blue Safe Stain (Life technologies), and seven sections for each gel lane were cut. Protein-containing gel pieces were washed with 100 μL of 0.1 M ammonium bicarbonate (5 min at RT). Then, 100 μL of 100% acetonitrile (ACN) was added to each tube, and the tubes were incubated for 5 min at RT. The liquid was discarded, the washing step repeated once more, and the gel plugs were shrunk by adding ACN. The dried gel pieces were reconstituted with 100 μL of 10 mM DTT/0.1 M ammonium bicarbonate and incubated for 40 min at 56 °C for cysteine reduction. The excess liquid was then discarded, and the cysteines were alkylated with 100 μL of 55 mM IAA/0.1 M ammonium bicarbonate (20 min at RT, in the dark). The liquid was discarded, the washing step was repeated once more, and the gel plugs were shrunk by adding ACN. The dried gel pieces were reconstituted with 12.5 ng/μL trypsin in 50 mM ammonium bicarbonate and digested overnight at 37 °C. The supernatant from the digestions was saved in a fresh tube, and 100 μL of 1% TFA/30% ACN was added to the gel pieces for an additional extraction of peptides. The extracted solution and digested mixture were then combined and vacuum-centrifuged for organic component evaporation. The peptides were resuspended with 40 μL of 2.5% ACN/0.1% TFA, desalted, filtered through a C18 microcolumn ZipTip, and eluted from the C18 bed using 10 μL of 80% ACN/0.1% TFA. The organic component was once again removed by evaporation in a vacuum centrifuge, and the peptides were resuspended in a suitable nanoLC injection volume (typically 3−10 μL) of 2.5% ACN/0.1% TFA.

### 4.3. NanoLC Analysis and Mass Spectrometry Analysis

An UltiMate 3000 RSLC nano-LC system (ThermoFisher Scientific, Waltham, MA, USA) equipped with an integrated nanoflow manager and microvacuum degasser was used for peptide separation. The peptides were loaded onto a 75 μm NanoSeries C18 column (ThermoFisher, P/N 164534) for multistep gradient elution (eluent A 0.05% TFA; eluent B 0.04% TFA in 80% ACN) from 5% to 20% eluent B within 10 min, from 20% to 50% eluent B within 45 min, and for further 5 min from 50% to 90% eluent B with a constant flow of 0.3 μL/min. After 5 min, the eluted sample fractions were continuously diluted with 1.2 μL/min a-cyano-4-hydroxycinnamic acid (CHCA) and spotted onto a MALDI target using a HTC-xt spotter (PAL SYSTEM) with an interval of 20 s resulting in 168 fractions for each gel slice. Mass Spectrometry Analysis MALDI-TOF MS spectra were acquired using a 5800 MALDI TOF/TOF Analyzer (Sciex, Concord, ON, Canada). The spectra were acquired in the positive reflector mode by 20 subspectral accumulations (each consisting of 50 laser shots) in an 800−4000 mass range, focus mass 2100 Da, using a 355 nm Nb:YAG laser with a 20 kV acceleration voltage. Peak labeling was automatically done by 4000 Series Explorer software Version 4.1.0 (Sciex) without any kind of smoothing of peaks or baseline, considering only peaks that exceeded a signal-to-noise ratio of 10 (local noise window 200 *m*/*z*) and a half maximal width of 2.9 bins. The calibration was performed using default calibration originated by five standard spots (Mass Standards kit for Calibration P/N 4333604). Only the MS/MS spectra of preselected peaks (out of peak pairs with a mass difference of 6.02, 10.01, 12.04, 16.03, and 20.02 Da) were integrated over 1000 laser shots in the 1 kV positive ion mode with the metastable suppressor turned on. Air at the medium gas pressure setting (1.25 × 10^−6^ Torr) was used as the collision gas in the CID-off mode. After smoothing and baseline subtractions, spectra were generated automatically by 4000 Series Explorer software. The MS and MS/MS spectra were processed by ProteinPilot Software 4.5 (Sciex) which acts as an interface between the Oracle database containing raw spectra and a local copy of the MASCOT search engine (Version 2.1, Matrix Science, Ltd.). The Paragon algorithm was used with identification as the Sample Type, iodacetamide as cysteine alkylation, with the search option “biological modifications” checked, and trypsin as the selected enzyme. MS/MS protein identification was performed against the Swiss-Prot database (number of protein sequences: 254757; released on 20121210) without taxon restriction using a confidence threshold of 95% (Proteinpilot Unused score ≥ 1.31). The monoisotopic precursor ion tolerance was set to 0.12 Da and the MS/MS ion tolerance to 0.3 Da. The minimum required peptide length was set to six amino acids.

### 4.4. Immunoblotting Analysis

Dilutions 1:10 of the serum samples were separated in 12% gels (Life technologies) by SDS-PAGE with MES running buffer (Life technologies) and electroblotted onto nitrocellulose (GE, Healthcare, Little Chalfont, UK) membranes. The blots were incubated with primary and secondary antibodies. The antibodies were revealed using ECL (Millipore). To control for equal protein loading and transfer, the membranes were stained with Ponceau S solution (Sigma). The following antibodies were used: anti-C-VTN (Abcam, Cambridge, UK, ab113700), anti-N-VTN (Santa Cruz Biotechnology, Dallas, Texas, USA, sc-15332); the secondary anti-rabbit peroxidase-conjugated antibody was from Jackson ImmunoResearch. The chemiluminescent blots were imaged with the ChemiDoc^TM^ Touch Imaging System (Bio-Rad Laboratories, Hercules, CA, USA), and vitronectin band densities were quantified by ImageLab software version 5.1.2 (Bio-Rad). Total protein lane densities by Ponceau S staining were used for normalization.

### 4.5. In Vitro Degradation of Vitronectin by Metalloproteases

MMP-2 (ab81550) and MMP-9 (ab82955) were from Abcam. Before incubation with the serum sample, pro MMP-9 was activated by incubation with 4-aminophenylmercuric acetate 1 mM (Sigma, St. Louis, MO, USA) overnight at 37 °C. The digestion of vitronectin was carried out by incubation of the substrate at 37 °C for 24 h with MMPs in an enzyme-to-substrate ratio of 1:20 in 50 mMTris-HCl, pH 7.5, containing 0.15 M NaCl, CaCl_2_ 10 mM, 0.05% Brij 35 and 0.02% NaN_3_.

### 4.6. Statistics

Outcome measures for between-group comparisons of patient characteristics (Age, body mass, AST, ALT, GGT, V10, and V75) were reported as mean and standard deviation or median and interquartile range, as appropriate. The Student t test or Mann–Whitney U test were used to compare groups for normally or not normally distributed data, respectively. The Pearson correlation coefficient or the Rho Spearman nonparametric correlation test were used. All tests are two-tailed, and a *p*-value of <0.05 was considered as statistically significant.

Linear multiple regression analyses were performed to identify factors independently associated with NAS and liver fibrosis. A further multiple logistic regression analysis was carried out to test the independent factors associated with the V10/V75 ratio above the upper normal value (0.27) found in healthy controls. The analyses were performed using SPSS software v.23 (IBM, Milan, Italy).

## Figures and Tables

**Table 1:** Clinical characteristics of NAFL and NASH populations.

	NAFL	NASH	*p* Value
All	27	23	
Gender (male/female)	14/13	10/13	0.55
Nas	2.81 ± 1.04	5.43 ± 0.99	**<0.001**
Fibrosis 1/2/3/4	11/12/4/0	3/5/9/6	**<0.001**
AGE at enrolment (years)	52.3 ± 14.4	51.7 ± .9.8	0.44
Body mass index (kg/m^2^)	28.7 ± 4.1	30.5 ± 3.9	0.067
AST (U/L)	37.6 ± 17.8	61.9 ± 40.9	**0.005**
ALT (U/L)	69.3 ± 36.1	97.1 ± 56.1	**0.024**
GGT (U/L)	62.4 ± 41.5	80.7 ± 86.7	0.18
AST/ALT ratio	0.56 ± 0.18	0.68 ± 0.31	0.07

Data are reported as Means ± Standard Deviation; *p* values <0.05 are reported in bold; AST (Aspartate transaminase); ALT (Alanine transaminase); GGT (Gamma-glutamyl transferase).

**Table 2:** ECM and ECM-associated components analysis of NASH fragments by MatrisomeDB 2.0.

Gene Symbol	Name	Matrisome Division	Category
*FGA*	Fibrinogen alpha chain	Core matrisome	ECM Glycoproteins
*VTN*	Vitronectin	Core matrisome	ECM Glycoproteins
*LGALS3*	Galectin-3	Matrisome-associated	ECM-affiliated Proteins
*LGALS4*	Galectin-4	Matrisome-associated	ECM-affiliated Proteins
*CTSB*	Cathepsin B	Matrisome-associated	ECM Regulators
*SERPINB1*	Leukocyte elastase inhibitor	Matrisome-associated	ECM Regulators
*SERPINC1*	Antithrombin-III	Matrisome-associated	ECM Regulators

## Supplementary Materials

Supplementary materials can be found at http://www.mdpi.com/1422-0067/19/2/603/s1.
